# Homopolymer and ABC Triblock Copolymer Mixtures for Thermoresponsive Gel Formulations

**DOI:** 10.3390/gels7030116

**Published:** 2021-08-09

**Authors:** Anna P. Constantinou, Nikitas Provatakis, Qian Li, Theoni K. Georgiou

**Affiliations:** 1 Department of Materials, Imperial College London, London SW7 2AZ, UK; anna.constantinou14@imperial.ac.uk (A.P.C.); qian.li16@imperial.ac.uk (Q.L.); 2 Department of Bioengineering, Imperial College London, London SW7 2AZ, UK; nikitasprovatakis@hotmail.com

**Keywords:** thermoresponsive gel, mixtures, triblock copolymer, homopolymers

## Abstract

Our group has recently invented a novel series of thermoresponsive ABC triblock terpolymers based on oligo(ethylene glycol) methyl ether methacrylate with average *M*_n_ 300 g mol^−1^ (OEGMA300, A unit), *n*-butyl methacrylate (BuMA, B unit) and di(ethylene glycol) methyl ether methacrylate (DEGMA, C unit) with excellent thermogelling properties. In this study, we investigate how the addition of OEGMA300_x_ homopolymers of varying molar mass (MM) affects the gelation characteristics of the best performing ABC triblock terpolymer. Interestingly, the gelation is not disrupted by the addition of the homopolymers, with the gelation temperature (*T_gel_*) remaining stable at around 30 °C, depending on the MM and content in OEGMA300_x_ homopolymer. Moreover, stronger gels are formed when higher MM OEGMA300_x_ homopolymers are added, presumably due to the homopolymer chains acting as bridges between the micelles formed by the triblock terpolymer, thus, favouring gelation. In summary, novel formulations based on mixtures of triblock copolymer and homopolymers are presented, which can provide a cost-effective alternative for use in biomedical applications, compared to the use of the triblock copolymer only.

## 1. Introduction

Stimulus-responsive polymers are polymers that respond to an external stimulus [1,2]. When this stimulus is temperature, the polymers are called thermoresponsive or temperature-responsive [3,4,5,6,7]. In some cases, when the temperature is varied, a polymer network is formed, which is called thermoresponsive gel. Of particular interest in the biomedical field are the lower-critical solution temperature (LCST) polymers, and, especially, the LCST thermoresponsive gels, which exist in a solution state at low temperature, but assemble into a physical three-dimensional (3D) network as the temperature rises [8,9,10,11,12]. This transition, known as sol–gel transition, should ideally occur between room temperature and body temperature to ensure that: (i) gelation will occur quickly at body temperature e.g., post injection, and (ii) gelation will not occur at room temperatures; thus, the sample can be easily injected. Thermoresponsive gels have received much attention and have been investigated as injectable gels in tissue engineering [3,12,13,14,15] and in drug delivery [16,17,18,19], and as 3D (bio-)printable materials [20,21,22,23,24,25].

Several studies have investigated the effect of additives on the thermoresponsive properties of the polymers, with major focus on Pluronic^®^ F127 (poloxamer 407) [26,27]. Pluronic^®^ polymers are ABA triblock copolymers, where A and B consist of ethylene glycol (EG) and propylene glycol (PG), respectively [12]. Several Pluronic^®^ polymers are commercially available differing in the total molar mass (MM) and the ratio of EG/PG. Pluronic^®^ F127 has an EG content of approximately 70% and a total MM of approximately 12600 g mol^−1^, and it has gained much attention due to its interesting thermogelling properties and commercial availability [12]. Polymeric additives in solutions of Pluronic^®^ F127, such as alginate [28,29,30], poly(ethylene glycol) (PEG) [27,31,32,33,34,35], poly(acrylic acid) (PAA) [27,36], and carbopols, i.e., crosslinked PAA [37], poly(vinyl alcohol) [27,38], and hyaluronic acid [39,40], have been investigated. Poloxamers as polymeric additives in solutions of Pluronic^®^ F127 have also been studied [19,41,42,43,44,45,46,47,48]. For example, mixtures of Pluronic^®^ F127 and Pluronic^®^ F68, EG content 80% and total MM 8350 g mol^−1^ [12], were tested for suitability in drug delivery [19,27,44,46,47,48,49,50]. In two of these studies, increasing the content in Pluronic^®^ F68 while keeping the content in Pluronic^®^ F127 constant lowered the gelation time at body temperature and drug diffusion kinetics were improved when this formulation was applied in drug delivery [46,47]. However, when the total polymer concentration was kept constant and the concentration of the two polymers was varied, it was observed that increasing the content in Pluronic^®^ F68 increased the gelation temperature (*T*_gel_) [49]. Finally, in other studies, the effect of the incorporation of solid lipid nanoparticles and carbopols in mixtures of Pluronic^®^ F127 with poloxamers on the gelation has also been investigated [41,42,43].

A few studies on mixtures of thermoresponsive polymers other than poloxamers have also been reported in the literature [51,52,53,54,55]. In two of the studies, mixtures of AB and BAB diblock and triblock copolymers, with A and B being based on EG and lactic acid, respectively, were investigated for thermoreversible gelation [52,53]. In these studies, the two polymers contained complementary chiral blocks of lactic acid; thus, gelation was favoured by stereocomplexation between the enantiomers [52,53]. Interestingly, when water-soluble and a water-insoluble poly(lactic acid-*co*-glycolic acid)-*b*-poly(ethylene glycol)-*b*-poly(lactic acid-*co*-glycolic acid) (PLGA–PEG–PLGA) triblock copolymers were mixed, this produced a new system that presented a wider gelation area than the one shown by the soluble copolymer itself [54]. In another study, mixtures of two triblock terpolymers, which independently exhibited thermoreversible gelation, were studied [51]. The polymers consisted of N-isopropylacrylamide (A unit), n-butyl acrylate (B unit), and *N*, *N*-dimethylacrylamide (C unit), and their structure was (A-*co*-B)-*b*-C-*b*-(A-*co*-B) and (A-*co*-B)-*b*-C-*b*-A. As suspected, the former gels formed at lower temperatures than the latter, which is attributed to the presence of additional hydrophobic *n*-butyl acrylate units in the third block. Interestingly, it has been clearly demonstrated that the *T*_gel_ increases linearly as the content in the (A-*co*-B)-*b*-C-*b*-A increases [51].

The studies discussed clearly demonstrate that the incorporation of additives in solutions of thermoresponsive gels can significantly impact the gelation parameters by affecting the underlying gelation mechanism. This suggests that there is scope in researching/developing additive incorporation as a potential method for optimising the *T*_gel_ and the gelation boundaries. In addition, most of the studies are focused on exploring the effects of incorporating polymeric additives in poloxamer solutions. However, many possibilities can be explored, and trends on how polymeric additives can impact the gelation of thermoresponsive polymers may be established.

Our group has recently reported a novel combination of methacrylate repeated units, which produces thermoresponsive polymers with a sharp sol–gel transition [56,57,58]. More specifically, the polymers consist of (i) the hydrophilic oligo (ethylene glycol) methyl ether methacrylate with average MM 300 g mol^−1^ (OEGMA300, A unit), which is also thermoresponsive at approximately 70 °C, depending on the MM [59], (ii) the hydrophobic n-butyl methacrylate (BuMA, B unit), and (iii) the hydrophilic and thermoresponsive (CP approximately 30 °C) [59] di(ethylene glycol) methyl ether methacrylate (DEGMA, C unit). Both ABC triblock copolymers of varying compositions and tetrablock terpolymers of varying architecture were investigated [57,58], with an ABC triblock terpolymer with OEGMA300-BuMA-DEGMA content at 40/35/25 *w/w*% showing promising thermogelation properties, with the gels being formed at concentrations as low as 2 *w/w*% [57]. Therefore, it is valuable to investigate the impact of polymeric additives on the gelation properties of this novel polymer.

To accomplish this task, we have performed a systematic investigation on mixtures of polymers by keeping the total polymer concentration constant at 20 *w/w*% in phosphate buffered saline (PBS), while varying the concentration ratio of the two polymers between 3:1, 1:1, and 1:3. A series of OEGMA300 homopolymers have been used as polymeric additives to investigate their impact on gelation properties: (i) OEGMA300_10_, (ii) OEGMA300_31_, (iii) OEGMA300_43_, and (iv) OEGMA300_75_. To analyse the impact of homopolymer additives on gelation and identify trends, we report the detailed phase diagrams and rheological properties of the mixtures at various concentrations and discuss the findings.

## 2. Results and Discussion

**Figure 1:**
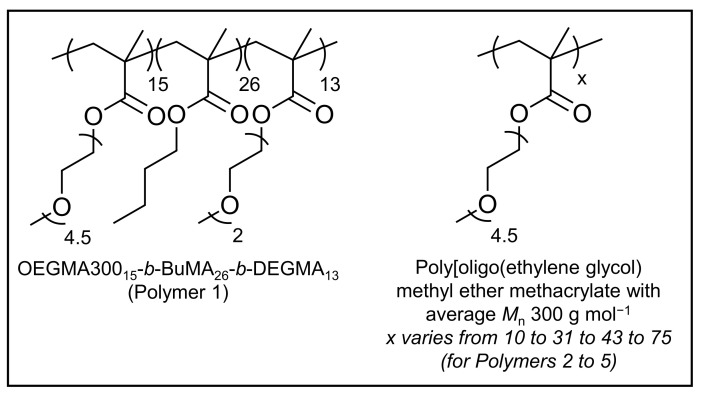
Chemical structures of the polymers; OEGMA300, BuMA, and DEGMA stand for oligo(ethylene glycol) methyl ether methacrylate with average *M*_n_ 300 g mol^−1^, *n*-butyl methacrylate, and di(ethylene glycol) methyl ether methacrylate, respectively.

As previously mentioned, five in-house synthesised PEG-based methacrylate polymers were used; specifically, one amphiphilic triblock terpolymer (OEGMA300_x_-*b*-BuMA_y_-*b*-DEGMA_z_, P1) and four OEGMA300 homopolymers of various MM values (poly(OEGMA300), P2 to P5) (Figure 1 and Table 1 (refer to Figure S1 for the GPC traces of the final polymers after precipitation)).

### 2.1. Homopolymers as Additives in Thermogelling Triblock Copolymer Solutions—Visual Phase Transitions

**Figure 2:**
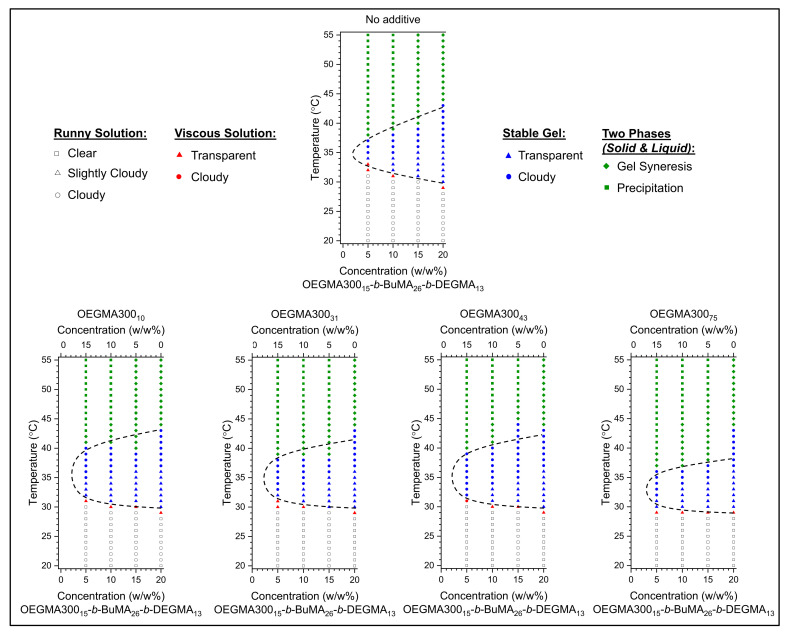
Phase diagrams in phosphate buffered saline (PBS) of mixtures of OEGMA300_15_-b-BuMA_26_-b-DEGMA_13_ (P1) without any additives (**top**), or by adding OEGMA300_x_ homopolymers of various degrees of polymerisation (**bottom**); when x = 10 (P2), x = 31 (P3), x = 43 (P4), and x = 75 (P5) from left to right. The mixtures were prepared at total polymer concentration 20 *w/w*% and a ratio of P1/OEGMA300_x_ equal to 1:3, 1:1, and 3:1. The following transitions are reported: (a) runny solution in white, (b) viscous solution in red, (c) stable gel in blue), and (d) phase separation in green. The gelation area is approximately indicated by a black dashed line.

To provide a basis of comparison, and to study how the phase transitions of the gelling agent are modified by the addition of homopolymers, it is necessary to construct the phase diagrams of the triblock copolymer in PBS without any additives (Figure 2 (top)). The following phases are reported: (a) runny solution state in white, (b) viscous solution state in red, (c) stable gel in blue, and (d) phase separation into solid and liquid in green. As demonstrated, the triblock copolymer presents a gelation area, which is approximately indicated by a black dashed line, close to body temperature. As has been well-documented, the gelation temperature (*T_gel_*) is tuned by the polymer concentration, i.e., *T_gel_* decreases as the concentration increases [3], and this is also observed for OEGMA300_15_-*b*-BuMA_26_-*b*-DEGMA_13_, with gelation being observed at the lowest concentration tested in this study, i.e., 5 *w/w*% in PBS. More specifically, OEGMA300_15_-*b*-BuMA_26_-*b*-DEGMA_13_ shows a smooth decrease in *T_gel_* from 34 to 30 °C as the concentration increases from 5 to 20 *w/w*%; thus, it is still a solution at room temperature. The gels destabilise when the temperature increases, either by precipitation (i.e., complete phase separation to solid and liquid) or gel syneresis (i.e., slight exclusion of solvent from the gel). This behaviour is advantageous as it shows that its gelation properties can be finely tuned to meet the properties of the targeted application.

To investigate the effect of OEGMA300 homopolymers on the thermogelling properties of OEGMA300_15_-*b*-BuMA_26_-*b*-DEGMA_13_ (Polymer 1), mixtures of OEGMA300_x_ with x varying from 10, 31, 43, and 75 with the triblock copolymer were investigated (Figure 2 (bottom) and Table 2). For this, the Polymer 1/OEGMA300_x_ ratio was targeted at 3:1, 1:1, and 1:3, with the total polymer concentration being kept constant at 20 *w/w*%.

It is worth noting that gelation is observed in all the mixtures, with both transparent and cloudy gels being formed. It is generally observed that the incorporation of OEGMA300_x_ keeps the *T_gel_* stable at values close to the *T_gel_* of 20 *w/w*% triblock copolymer solution (equal to 30 °C), regardless of the content or MM of OEGMA300_x_. This suggests that OEGMA300_x_ polymers might act as bridges for connecting the micelles formed by the amphiphilic triblock terpolymer, thus favouring gelation. This is in contrast with what has been observed for Pluronic^®^ F127, whose gelation is disrupted when PEG is added [32,33]. This is advantageous, as cost-effective formulations may be produced when OEGMA300_15_-*b*-BuMA_26_-*b*-DEGMA_13_ is mixed with OEGMA300_x_, due to the easier and cheaper production of the homopolymer, i.e., one synthetic step and one monomer needed for its synthesis.

Gel syneresis, followed by precipitation, is observed when the mixtures are heated above their gelation points. Interestingly, when the content in the triblock copolymer increases and, thus, the content in OEGMA300_x_ decreases, the precipitation temperature (T_prec_) increases, thus indicating an increased stability of those gel formulations. It is also worth noting that, when the concentration of OEGMA300_10_ is kept at low values (5 *w/w*%), the transitions of the mixture (15 *w/w*% P1 and 5 *w/w*% OEGMA300_10_) are almost identical to the ones observed when 15 *w/w*% P1 was tested. This indicates that, when the content in low MM OEGMA300 is kept at low values, the visual phase transitions are not affected.

### 2.2. Homopolymers as Additives in Thermogelling Triblock Copolymer Solutions—Rheological Properties

**Figure 3:**
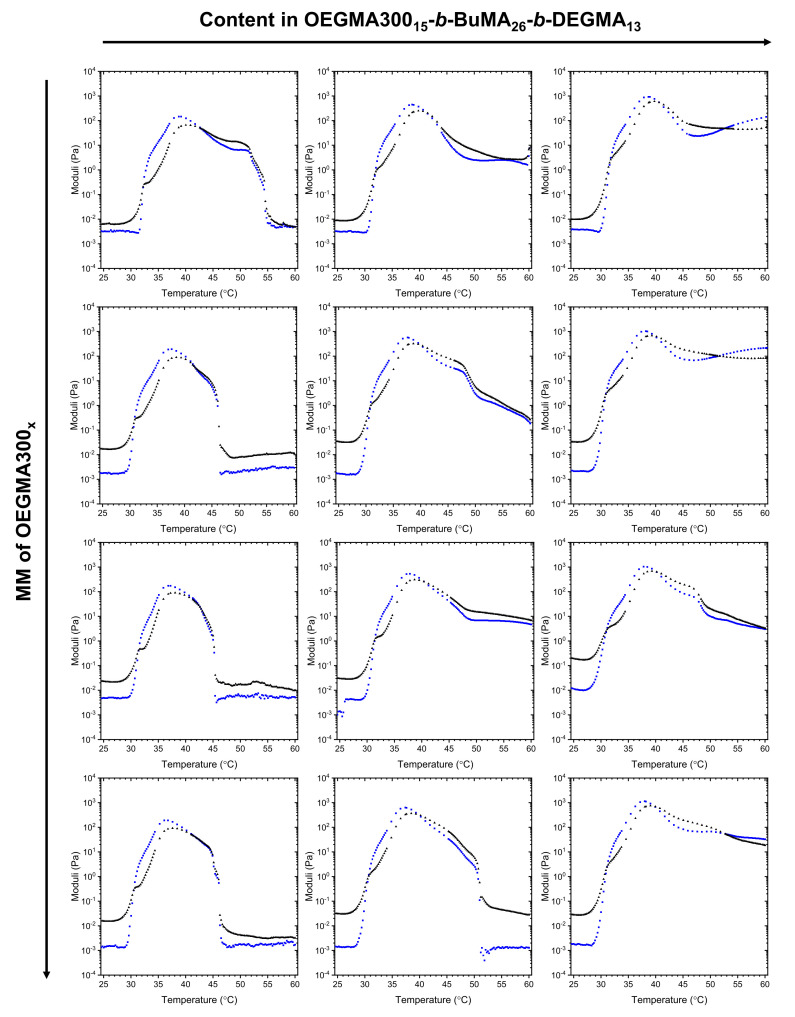
Storage modulus (G’, in blue squares) and loss modulus (G´´, in black triangles) as a function of temperature. The formulations consisted of 20 *w/w*% total polymer concentration in phosphate buffered saline, and the ratio of P1/OEGMA300_x_ was kept equal to 1:3, 1:1, and 3:1; this is shown from left to right. The MM of OEGMA300_x_ homopolymers was also varied from 10, 31, 43, to 75, shown from top to bottom.

To confirm the trends observed by visual tests, and to investigate the effect of the additives on the gel strength, rheological studies were also performed, and the results are present in Figure 3 and Figure S3. As can be seen in all the cases, a stable gel was formed, i.e., G’ > G’’, as the temperature increased to around 30 to 32 °C, which agrees with the results obtained by visual tests. As the temperature increases further, the strength of the gel increases, as the transparent gels become cloudy. A second crossover is observed at temperatures close to the ones at which gel syneresis and precipitation are detected visually. In some cases, where the concentration of P1 in the mixture is at 15 *w/w*% (P1:OEGMA300_x_ = 3:1), a second crossover (G’ > G’’) is observed by rheology, while, by visual tests, gel syneresis is observed, followed by precipitation at higher temperatures. At this point, it should be reminded that gel syneresis is a phenomenon first reported by Graham, and it is defined as the disturbance of the gel due to internat stresses, leading to slight exclusion of solvent [60,61]. On the other hand, precipitation is phase separation, which, in this study, is recorded as the start point of precipitation, and might proceed as the temperature increases further, until complete phase separation, i.e., solid polymer and liquid solvent, takes place. It is speculated that, upon shear stress, rearrangement of the polymer chains might take place; thus, the polymer network is reformed by rheology. This is observed at high contents in P1, as the concentrated solutions (15 and 20 *w/w*%) of the triblock terpolymer in PBS only present gel syneresis upon temperature increase, with no precipitation being detected, i.e., the gel does not progressively exclude more solvent. This double crossover has been previously observed by our group on OEGMA300_x_-*b*-BuMA_y_-*b*-DEGMA_z_-*b*-OEGMA300_x_ tetrablock terpolymer [58].

**Figure 4:**
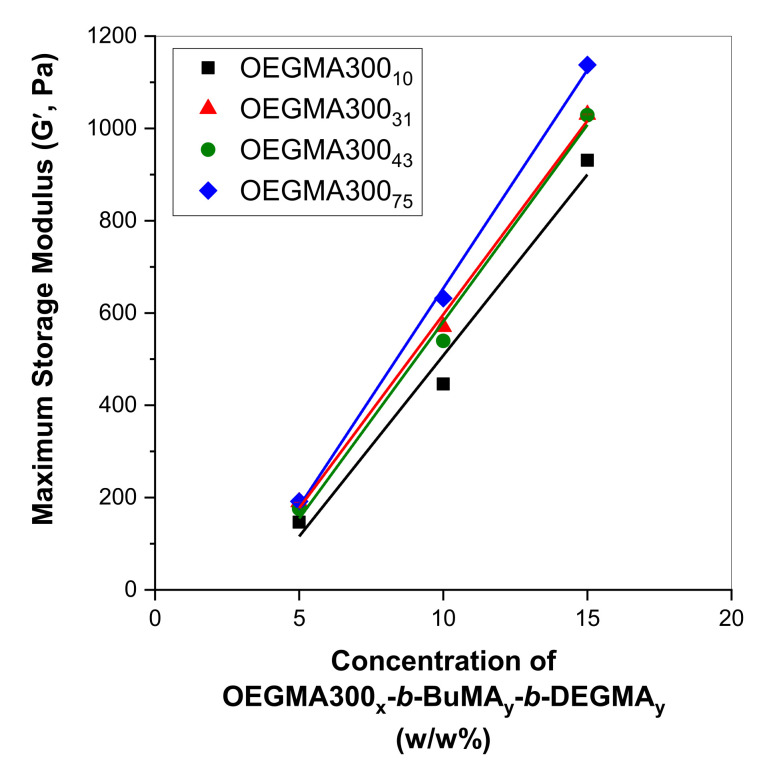
Maximum storage modulus (G´) as a function of the concentration of the triblock terpolymer, in mixtures with OEGMA300_x_ homopolymers. The total concentration was kept at 20 *w/w*% in phosphate buffered saline (PBS). The degree of polymerisation of the OEGMA300_x_ homopolymers was varied from 10 (black squares), to 31 (red triangles), to 43 (green circles), to 75 (blue rhombi). The data were fit with linear function.

The MM of the OEGMA300_x_ homopolymers plays a critical role in the stability of the gels, as the higher the MM, the higher the maximum storage modulus of the gels (Figure 4 from black (OEGMA300_10_) to red/green (OEGMA300_31_/OEGMA300_47_) to blue (OEGMA300_75_)). This can be explained, as the increase in MM might facilitate the formation of stronger entanglements between the well-hydrated coronas of the micelles, and, thus, favours the formation of a stronger gel network, i.e., interconnected micelles. However, the higher the MM, precipitation, i.e., complete phase separation, is favoured more, as indicated by the sudden drop in the magnitude of the moduli (Figure 3 (top to bottom)). This confirms the results obtained by visual tests, in which the addition of low MM OEGMA300_x_ at low concentrations does not affect the gelation properties of the triblock terpolymer significantly, while, when high MM OEGMA300_x_ are used as additives, gel destabilisation, and, thus, precipitation, is favoured.

When the ratio of P1 to OEGMA300_x_ is varied from 1:3, 1:1, to 3:1, the maximum storage modulus increases (Figure 4). This is expected. As the concentration of the gelling agent increases, the number of micelles increases; thus, more junction points are formed within the gel structure, leading to a stronger gel. This is a well-established phenomenon, and it has been previously observed in solutions consisting of a single gelling agent, such as methacrylate polymers [57,62] and poly(N-isopropylacrylamide)-based polymers [63,64].

## 3. Conclusions

In this study, the thermogelling properties of polymeric mixtures are investigated and compared to the corresponding polymer solutions without any polymeric additives. The thermogelling agent used is an ABC triblock terpolymer, namely, OEGMA300_15_-*b*-BuMA_26_-*b*-DEGMA_13_ (P1), while OEGMA300_x_ homopolymers of various MMs were used as additives: OEGMA300_10_ (P2), OEGMA300_31_ (P3), OEGMA300_43_ (P4), and OEGMA300_75_ (P5). The mixtures were investigated at a total polymer concentration of 20 *w/w*% in PBS, and concentration ratio of P1 to OEGMA300_x_ equal to 1:1, 3:1, and 1:3. It is demonstrated that the gelation properties of the triblock terpolymer are preserved when OEGMA300_x_ homopolymers are added. Interestingly, the *T*_gel_ is balanced at around 30 to 32 °C, which is the *T*_gel_ of 20 *w/w*% solution of the gelling agent, in the absence of additives. It is also shown that the addition of low MM OEGMA300_x_ homopolymers does not significantly affect the gelation properties of the pristine triblock terpolymer, while using higher MM OEGMA300_x_ homopolymers produces stronger gels, as the polymer chains of the homopolymers might act as bridges connecting the micelles. This study demonstrates that alternative cost-effective formulations can be produced, with the MM of the OEGMA300_x_ homopolymer playing a critical role in the stability and strength of the gels.

## 4. Materials and Methods

### 4.1. Materials

Sigma Aldrich Ltd., Saint Louis, MO, USA, was the provider phosphate buffered saline (PBS) tablets, while phosphate buffer saline (PBS 10 solution) was purchased from Fischer Scientific UK Ltd., Loughborough, UK. The methacrylate polymers, i.e., the four OEGMA300 homopolymers of varying MM values, and the thermogelling triblock terpolymer, OEGMA300_x_-*b*-BuMA_y_-*b*-DEGMA_z_ were in-house synthesised, via group transfer polymerisation (GTP), with their detailed synthesis reported elsewhere [57,59]. The structural properties of the in-house synthesised polymers have been determined via gel permeation chromatography (GPC) and proton nuclear magnetic resonance (^1^H NMR) spectroscopy, but discussion of the synthesis is out of the scope of this study.

### 4.2. Methods

#### 4.2.1. Sample Preparation for Visual Tests and Rheological Measurements

Concentrated (20 *w/w*%) stock solutions of the polymers in PBS were prepared, and they were used to prepare the mixtures, as described below. The triblock copolymer solutions (additive 1) were mixed with solutions of OEGMA300 homopolymers of various MM values (additive 2, Polymers 2 to 5), while keeping the total polymer concentration constant at 20 *w/w*%. The mixtures were prepared with concentration ratio of additive 1:additive 2 equal to 1:3, 1:1, and 3:1, e.g., 5 *w/w*% additive 1 and 15 *w/w*% additive 2 for ratio 1:3. Solutions of the triblock copolymer in PBS were also prepared for comparison purposes at concentrations equal to 5, 10, 15, and 20 *w/w*%.

#### 4.2.2. Visual Tests

An IKA RCT basic stirrer hotplate, an IKA ETS-D5 temperature controller, and a continuously stirred water bath were used to optically determine the changes of the aqueous polymer solutions. The visual tests were performed in the 20 to 80 °C range, and a visual change was recorded every 1 °C. The observations were determined by visual inspection, i.e., by eye, and notes were taken. The gelation temperature, *T*_gel_, was of foremost interest, and it was determined by the tube inversion method, i.e., no sample flow was detected upon tube inversion, indicating the formation of a stable gel. Several additional phases were detected and recorded, as follows: (i) runny solutions (clear, slightly cloudy, and cloudy), (ii) viscous solution (transparent and cloudy), (iii) stable gel (transparent and cloudy), and (iv) phase separation into insoluble solid and supernatant liquid (gel syneresis and precipitation). For more details regarding these transitions, please refer to Figure 7 (bottom right) in our previous publication [65].

#### 4.2.3. Rheology

The rheological properties of the polymer solutions were recorded using a TA Discovery HR-1 hybrid rheometer (TA Instruments, U.K.). The experiments were performed using a 40 mm Peltier steel plate (996921) and a solvent trap. The samples were subjected in temperature ramp measurements, during which the angular frequency (ω) and strain (γ) were kept constant at 1 rad s^−1^ and 1%, respectively. These values were chosen according to preliminary studies. The strain was kept at low values to ensure that it was within the linear viscoelastic area of the gel, i.e., if the strain is high enough, it can break the physical bonds of the polymer network. The value of angular viscosity was chosen to be at 1 rad s^−1^ to ensure that the sample behaves as a liquid at room temperature, while forming a gel at higher temperatures. The gel point by rheology was determined as the temperature at which the storage modulus (elastic modulus, G’) exceeded the loss modulus (viscous modulus, G”).

## 5. Patents

The novel combination of repeated units in OEGMA300_x_-*b*-BuMA_y_-*b*-DEGMA_z_ has been patented by A.P.C. and T.K.G. [56].

## Figures and Tables

**Table 1:** Experimental polymer structures, experimental molar mass (number—average molar mass, *M*_n_), and dispersity indices, where available.

No.	Experimental Polymer Structure *	*M*_n_ (g mol^−1^)	*Ð*
1	OEGMA300_15_-*b*-BuMA_26_-*b*-DEGMA_13_	10,400	1.15
2	OEGMA300_10_	3000	1.19
3	OEGMA300_31_	9440	1.17
4	OEGMA300_43_	13,000	1.18
5	OEGMA300_75_	22,400	1.23

* The experimental polymer structures denote the experimental degrees of polymerisation, as calculated by using the experimental molar mass and composition values, resulted by gel permeation chromatography (GPC) (in tetrahydrofuran using poly(methyl methacrylate) standard samples) and proton nuclear magnetic resonance (^1^H NMR) spectroscopy, respectively.

**Table 2:** Important phase transitions recorded by visual tests in phosphate buffered saline (PBS): gelation temperature (*T*_gel_) and temperatures at which gel syneresis (*T*_syn_) and precipitation (*T*_prec_) were observed.

Content in P1 (*w/w*%)	Content in OEGMA300_x_ (*w/w*%)	*T_gel_* (±2 °C)	*T_syn_* (±2 °C)	*T_prec_* (±2 °C)
5	0	34	38	42
10	0	32	39	42
15	0	31	40	----*
20	0	30	44	----*
**OEGMA300_10_ (P2)**				
5	15	32	41	43
10	10	31	41	45
15	5	31	40	72
**OEGMA300_31_ (P3)**				
5	15	32	39	41
10	10	31	39	45
15	5	30	39	48
**OEGMA300_43_ (P4)**				
5	15	32	40	42
10	10	31	41	45
15	5	31	45	49
**OEGMA300_75_ (P5)**				
5	15	30	37	40
10	10	30	37	45
15	5	30	38	50

* No precipitation was observed up to the highest temperature tested, i.e., 80 °C.

## Supplementary Materials

The following are available online at https://www.mdpi.com/article/10.3390/gels7030116/s1, Figure S1: GPC traces of the final polymers after precipitation. OEGMA300 homopolymers of varying degrees of polymerisation are shown in blue—OEGMA300_10_ in dark blue, OEGMA300_31_ in blue, OEGMA300_43_ in light blue, and OEGMA300_75_ in faded blue. The triblock terpolymer is shown in dark red line. Figure S2: Phase diagram of the mixture of OEGMA300_15_-*b*-BuMA_26_-*b*-DEGMA_13_ with OEGMA300_10_ in phosphate buffered saline (PBS) with transitions showing up to 80 °C. Figure S3: Temperature ramp rheological measurement on the mixture of OEGMA300_15_-*b*-BuMA_26_-*b*-DEGMA_13_ with OEGMA300_10_ at a ratio of 3:1 in phosphate buffered saline (PBS) with transitions showing up to 80 °C—storage modulus in blue squares and loss modulus in black triangles.

## References

[1] Pasparakis G., Vamvakaki M. (2011). Multiresponsive polymers: Nano-sized assemblies, stimuli-sensitive gels and smart surfaces. Polym. Chem..

[2] Manouras T., Vamvakaki M. (2017). Field responsive materials: Photo-, electro-, magnetic- and ultrasound-sensitive polymers. Polym. Chem..

[3] Constantinou A.P., Georgiou T.K. (2016). Tuning the gelation of thermoresponsive gels. Eur. Polym. J..

[4] Klouda L., Mikos A.G. (2008). Thermoresponsive hydrogels in biomedical applications. Eur. J. Pharm. Biopharm..

[5] Klouda L. (2015). Thermoresponsive hydrogels in biomedical applications: A seven-year update. Eur. J. Pharm. Biopharm..

[6] Constantinou A.P., Georgiou T.K., Khutoryanskiy V.V., Georgiou T.K. (2018). Thermoresponsive multiblock copolymers: Chemistry, properties and applications. Temperature-Responsive Polymers: Chemistry, Properties, and Applications.

[7] Nele V., Wojciechowski J.P., Armstrong J.P.K., Stevens M.M. (2020). Tailoring gelation mechanisms for advanced hydrogel applications. Adv. Funct. Mater..

[8] Southall N.T., Dill K.A., Haymet A.D.J. (2002). A view of the hydrophobic effect. J. Phys. Chem. B.

[9] Foster J.C., Akar I., Grocott M.C., Pearce A.K., Mathers R.T., O’Reilly R.K. (2020). 100th anniversary of macromolecular science viewpoint: The role of hydrophobicity in polymer phenomena. ACS Macro Lett..

[10] Palmese L.L., Thapa R.K., Sullivan M.O., Kiick K.L. (2019). Hybrid hydrogels for biomedical applications. Curr. Opin. Chem. Eng..

[11] Cook M.T., Haddow P., Kirton S.B., McAuley W.J. (2021). Polymers exhibiting lower critical solution temperatures as a route to thermoreversible gelators for healthcare. Adv. Funct. Mater..

[12] Constantinou A.P., Georgiou T.K. (2021). Pre-clinical and clinical applications of thermoreversible hydrogels in biomedical engineering: A review. Polym. Int..

[13] Place E.S., George J.H., Williams C.K., Stevens M.M. (2009). Synthetic polymer scaffolds for tissue engineering. Chem. Soc. Rev..

[14] Gutowska A., Jeong B., Jasionowski M. (2001). Injectable gels for tissue engineering. Anat. Rec..

[15] Ruel-Gariépy E., Leroux J. (2004). In situ-forming hydrogels—review of temperature-sensitive systems. Eur. J. Pharm. Biopharm..

[16] Bobbala S., Tamboli V., McDowell A., Mitra A.K., Hook S. (2016). Novel injectable pentablock copolymer based thermoresponsive hydrogels for sustained release vaccines. AAPS J..

[17] Xie B., Jin L., Luo Z., Yu J., Shi S., Zhang Z., Shen M., Chen H., Li X., Song Z. (2015). An injectable thermosensitive polymeric hydrogel for sustained release of Avastin1 to treat posterior segment disease. Int. J. Pharm..

[18] Xi L., Wang T., Zhao F., Zheng Q., Li X., Luo J., Liu J., Quan D., Ge J. (2014). Evaluation of an injectable thermosensitive hydrogel as drug delivery implant for ocular glaucoma surgery. PLoS ONE.

[19] Xuan J.-J., Balakrishnan P., Oh D.H., Yeo W.H., Park S.M., Yong C.S., Choi H.-G. (2010). Rheological characterization and in vivo evaluation of thermosensitive poloxamer-based hydrogel for intramuscular injection of piroxicam. Int. J. Pharm..

[20] Feilden E., Ferraro C., Zhang Q., García-Tuñón E., D’Elia E., Giuliani F., Vandeperre L., Saiz E. (2017). 3D printing bioinspired ceramic composites. Sci. Rep..

[21] Rocha V.G., García-Tuñón E., Botas C., Markoulidis F., Feilden E., D’Elia E., Ni N., Shaffer M., Saiz E. (2017). Multimaterial 3D printing of graphene-based electrodes for electrochemical energy storage using thermoresponsive inks. ACS Appl. Mater. Interfaces.

[22] Feilden E., Blanca E.G., Giuliani F., Saiz E., Vandeperre L. (2016). Robocasting of structural ceramic parts with hydrogel inks. J. Eur. Ceram. Soc..

[23] Zhang M., Vora A., Han W., Wojtecki R.J., Maune H., Le A.B.A., Thompson L.E., McClelland G.M., Ribet F., Engler A.C. (2015). Dual-responsive hydrogels for direct-write 3D printing. Macromolecules.

[24] Bakirci E., Toprakhisar B., Zeybek M.C., Ince G.O., Koc B. (2017). Cell sheet based bioink for 3D bioprinting applications. Biofabrication.

[25] Müller M., Becher J., Schnabelrauch M., Zenobi-Wong M. (2015). Nanostructured pluronic hydrogels as bioinks for 3D Bioprinting. Biofabrication.

[26] Abou-Shamat M., Calvo-Castro J., Stair J.L., Cook M.T. (2019). Modifying the properties of thermogelling poloxamer 407 solutions through covalent modification and the use of polymer additives. Macromol. Chem. Phys..

[27] Abou-Shamat M., Stair J.L., Kirton S.B., Calvo-Castro J., Cook M.T. (2020). A design-of-experiments approach to developing thermoresponsive gelators from complex polymer mixtures. Mol. Syst. Des. Eng..

[28] Grassi G., Crevatin A., Farra R., Guarnieri G., Pascotto A., Rehimers B., Lapasin R., Grassi M. (2006). Rheological properties of aqueous pluronic–alginate systems containing liposomes. J. Colloid Interface Sci..

[29] Lin H., Sung K.C., Vong W. (2004). In situ gelling of alginate/pluronic solutions for ophthalmic delivery of pilocarpine. Biomacromolecules.

[30] Chen C., Fang C., Al-Suwayeh S.A., Leu Y., Fang J. (2011). Transdermal delivery of selegiline from alginate–pluronic composite thermogels. Int. J. Pharm..

[31] Malmsten M., Lindman B. (1993). Effects of homopolymers on the gel formation in aqueous block copolymer solutions. Macromolecules.

[32] Ricardo N.M.P.S., Ricardo N.M.P.S., Costa F.D.M.L.L., Bezerra F.W.A., Chaibundit C., Hermida-Merino D., Greenland B.W., Burattini S., Hamley I.W., Keith N.S. (2012). Effect of water-soluble polymers, polyethylene glycol and poly (vinylpyrrolidone), on the gelation of aqueous micellar solutions of pluronic copolymer F127. J. Colloid Interface Sci..

[33] Pragatheeswaran A.M., Chen S.B. (2013). Effect of chain length of PEO on the gelation and micellization of the pluronic F127 copolymer aqueous system. Langmuir.

[34] Gilbert J.C., Richardson J.L., Davies M.C., Palin K.J., Hadgraft J. (1987). The effect of solutes and polymers on the gelation properties of pluronic F-127 solutions for controlled drug delivery. J. Control. Release.

[35] Colly A., Marquette C., Courtial E. (2021). Poloxamer/poly (ethylene glycol) self-healing hydrogel for high-precision freeform reversible embedding of suspended hydrogel. Langmuir.

[36] Pragatheeswaran A.M., Chen S.B. (2016). The influence of poly (acrylic acid) on micellization and gelation characteristics of aqueous pluronic F127 copolymer system. Colloid. Polym. Sci..

[37] Jones D.S., Bruschi M.L., de Freitas O., Gremião M.P.D., Lara E.H.G., Andrews G.P. (2009). Rheological, mechanical and mucoadhesive properties of thermoresponsive, bioadhesive binary mixtures composed of poloxamer 407 and carbopol 974P designed as platforms for implantable drug delivery systems for use in the oral cavity. Int. J. Pharm..

[38] Bercea M., Darie R.N., Niţă L.E., Morariu S. (2011). Temperature responsive gels based on pluronic F127 and poly (vinyl alcohol). Ind Eng. Chem. Res..

[39] Mayol L., Quaglia F., Borzacchiello A., Ambrosio L., Rotonda M.I.L. (2008). A novel poloxamers/hyaluronic acid in situ forming hydrogel for drug delivery: Rheological, mucoadhesive and in vitro release properties. Eur. J. Pharm. Biopharm..

[40] Nascimento M.H.M., Franco M.K.K.D., Yokaichyia F., de Paula E., Lombello C.B., de Araujo D.R. (2018). Hyaluronic acid in pluronic F-127/F-108 hydrogels for postoperative pain in arthroplasties: Influence on physico-chemical properties and structural requirements for sustained drug-release. Int. J. Biol. Macromol..

[41] Din F.U., Mustapha O., Kim D.W., Rashid R., Park J.H., Choi J.Y., Ku S.K., Yong C.S., Kim J.O., Choi H. (2015). Novel dual-reverse thermosensitive solid lipid nanoparticle-loaded hydrogel for rectal administration of flurbiprofen with improved bioavailability and reduced initial burst effect. Eur. J. Pharm. Biopharm..

[42] Majithiya R.J., Ghosh P.K., Umrethia M.L., Murthy R.S.R. (2006). Thermoreversible-mucoadhesive gel for nasal delivery of sumatriptan. AAPS PharmSciTech.

[43] Ryu J., Chung S., Lee M., Kim C., Shim C.K. (1999). Increased bioavailability of propranolol in rats by retaining thermally gelling liquid suppositories in the rectum. J. Control. Release.

[44] Baldassari S., Solari A., Zuccari G., Drava G., Pastorino S., Fucile C., Marini V., Daga A., Pattarozzi A., Ratto A. (2018). Development of an injectable slow-release metformin formulation and evaluation of its potential antitumor effects. Sci. Rep..

[45] Yu Z., Guo F., Guo Y., Zhang Z., Wu F., Luo X. (2017). Optimization and evaluation of astragalus polysaccharide injectable thermoresponsive in-situ gels. PLoS ONE.

[46] Xuan J., Yan Y., Oh D.H., Choi Y.K., Yong C.S., Choi H. (2011). Development of thermo-sensitive injectable hydrogel with sustained release of doxorubicin: Rheological characterization and in vivo evaluation in rats. Drug Deliv..

[47] Choi H., Jung J., Ryu J., Yoon S., Oh Y., Kim C. (1998). Development of in situ-gelling and mucoadhesive acetaminophen liquid suppository. Int. J. Pharm..

[48] Mao Y., Li X., Chen G., Wang S. (2016). Thermosensitive hydrogel system with paclitaxel liposomes used in localized drug delivery system for in situ treatment of tumor: Better antitumor efficacy and lower toxicity. J. Pharm. Sci..

[49] Al Khateb K., Ozhmukhametova E.K., Mussin M.N., Seilkhanov S.K., Rakhypbekov T.K., Lau W.M., Khutoryanskiy V.V. (2016). In situ gelling systems based on pluronic F127/pluronic F68 formulations for ocular drug delivery. Int. J. Pharm..

[50] Kim E., Gao Z., Park J., Li H., Han K. (2002). rhEGF/HP-Β-CD complex in poloxamer gel for ophthalmic delivery. Int. J. Pharm..

[51] Onoda M., Ueki T., Tamate R., Akimoto A.M., Hall C.C., Lodge T.P., Yoshida R. (2018). Precisely tunable sol–gel transition temperature by blending thermoresponsive ABC triblock terpolymers. ACS Macro Lett..

[52] Mao H., Pan P., Shan G., Bao Y. (2015). In situ formation and gelation mechanism of thermoresponsive stereocomplexed hydrogels upon mixing diblock and triblock poly (lactic acid)/poly (ethylene glycol) copolymers. J. Phys. Chem. B.

[53] Mao H., Wang C., Chang X., Cao H., Shan G., Bao Y., Pan P. (2018). Poly (lactic acid)/poly (ethylene glycol) stereocomplexed physical hydrogels showing thermally-induced gel–sol–gel multiple phase transitions. Mater. Chem. Front..

[54] Li K., Yu L., Liu X., Chen C., Chen Q., Ding J. (2013). A long-acting formulation of a polypeptide drug exenatide in treatment of diabetes using an injectable block copolymer hydrogel. Biomaterials.

[55] Zhang B., Xie S., Lodge T.P., Bates F.S. (2021). Phase behavior of diblock copolymer–homopolymer ternary blends with a compositionally asymmetric diblock copolymer. Macromolecules.

[56] Georgiou T., Constantinou A. Polymers. 2020, PCT/GB20 19/052686. https://patentimages.storage.googleapis.com/cd/31/58/ef3390d5313a8b/WO2020065295A1.pdf.

[57] Constantinou A.P., Zhan B., Georgiou T.K. (2021). Tuning the gelation of thermoresponsive gels based on triblock terpolymers. Macromolecules.

[58] Constantinou A.P., Zhang K., Somuncuoğlu B., Feng B., Georgiou T.K. (2021). PEG-based methacrylate tetrablock terpolymers: How does the architecture control the gelation?. Macromolecules.

[59] Li Q., Constantinou A.P., Georgiou T.K. (2021). A library of thermoresponsive PEG-based methacrylate homopolymers: How do the molar mass and number of ethylene glycol groups affect the cloud point?. J. Polym. Sci..

[60] Kunitz M. (1928). Syneresis and swelling of gelatin. J. Gen. Physiol..

[61] Van Dijk H.J.M., Walstra P., Schenk J. (1984). Theoretical and experimental study of one-dimensional syneresis of a protein gel. Chem. Eng. J..

[62] Constantinou A.P., Patias G., Somuncuoğlu B., Brock T., Lester D.W., Haddleton D.M., Georgiou T.K. (2021). Homo-and co-polymerisation of di (propylene glycol) methyl ether methacrylate–a new monomer. Polym. Chem..

[63] Haddow P., McAuley W.J., Kirton S.B., Cook M.T. (2020). Poly (N-isopropyl acrylamide)–poly (ethylene glycol)–poly (N-isopropyl acrylamide) as a thermoreversible gelator for topical administration. Mater. Adv..

[64] Kirkland S.E., Hensarling R.M., McConaughy S.D., Guo Y., Jarrett W.L., McCormick C.L. (2008). Thermoreversible hydrogels from RAFT-synthesized BAB triblock copolymers: Steps toward biomimetic matrices for tissue regeneration. Biomacromolecules.

[65] Constantinou A.P., Zhao H., McGilvery C.M., Porter A.E., Georgiou T.K. (2017). A Comprehensive systematic study on thermoresponsive gels: Beyond the common architectures of linear terpolymers. Polymers.

