# Supplementary material for: Homopolymer and ABC Triblock Copolymer Mixtures for Thermoresponsive Gel Formulations
Source: Gels. 2021 Aug 9;7(3):116. doi: 10.3390/gels7030116 (PMC8395906; doi:10.3390/gels7030116)
Supplement: Supplementary file 1 [file gels-07-00116-s001.zip › gels-1318238-Supplementary-for publish.pdf]

gels

MDPI

Homopolymers and ABC Triblock Copolymer Mixtures for Thermoresponsive gel Formulations

Anna P. Constantinou, Nikitas Provatakis, Qian Li and Theoni K. Georgiou*

Supplementary Information

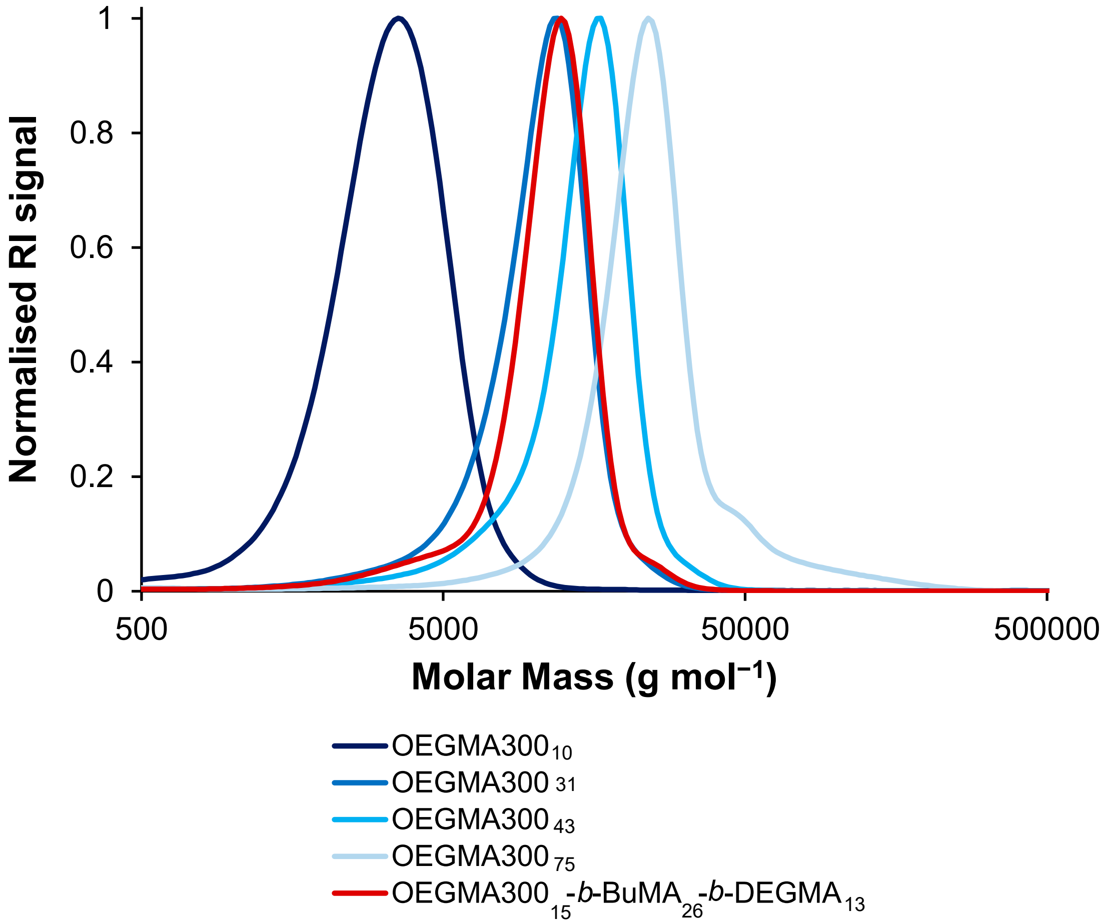

Figure S1. GPC traces of the final polymers after precipitation. OEGMA300 homopolymers of varying degrees of polymerisation are shown in blue: $OEGMA300_{10}$ in dark blue, $OEGMA300_{31}$ in blue, $OEGMA300_{43}$ in light blue, and $OEGMA300_{75}$ in faded blue. The triblock terpolymer is shown in dark red line.

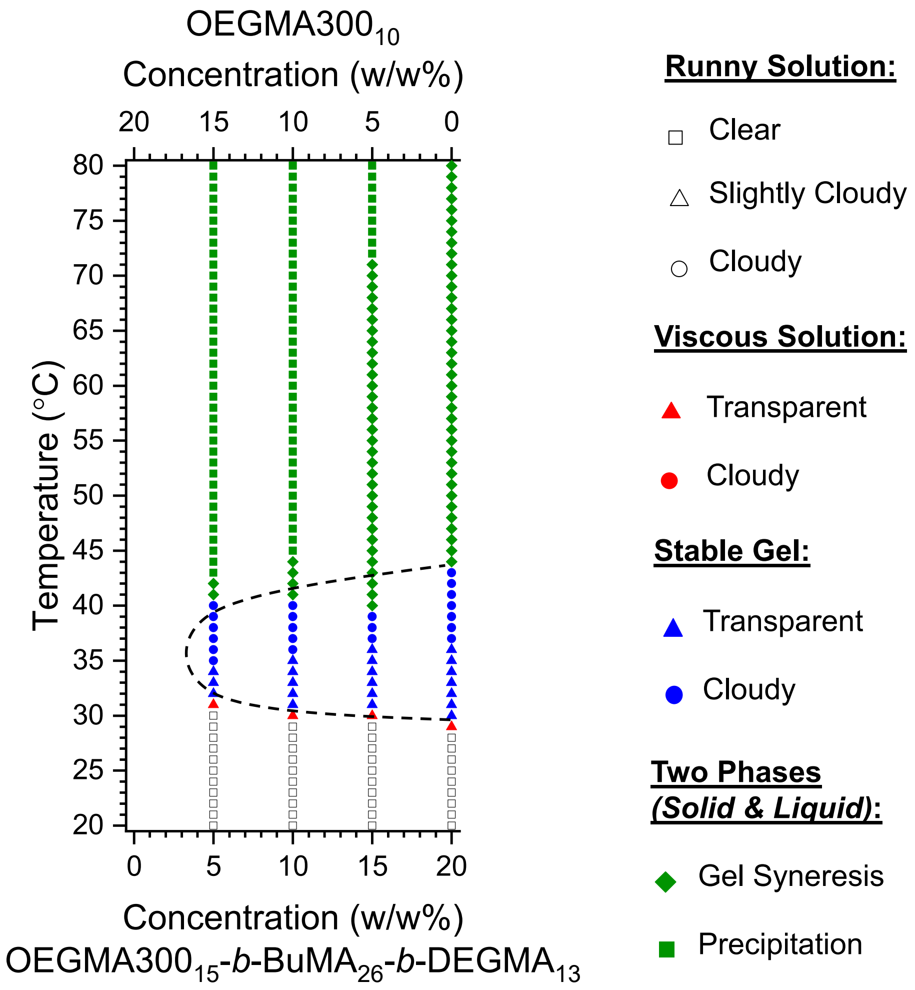

Figure S2. Phase diagram of the mixture of OEGMA300$_{15}$-*b*-BuMA$_{26}$-*b*-DEGMA$_{13}$ with OEGMA300$_{10}$ in phosphate buffered saline (PBS) with transitions showing up to 80 °C.

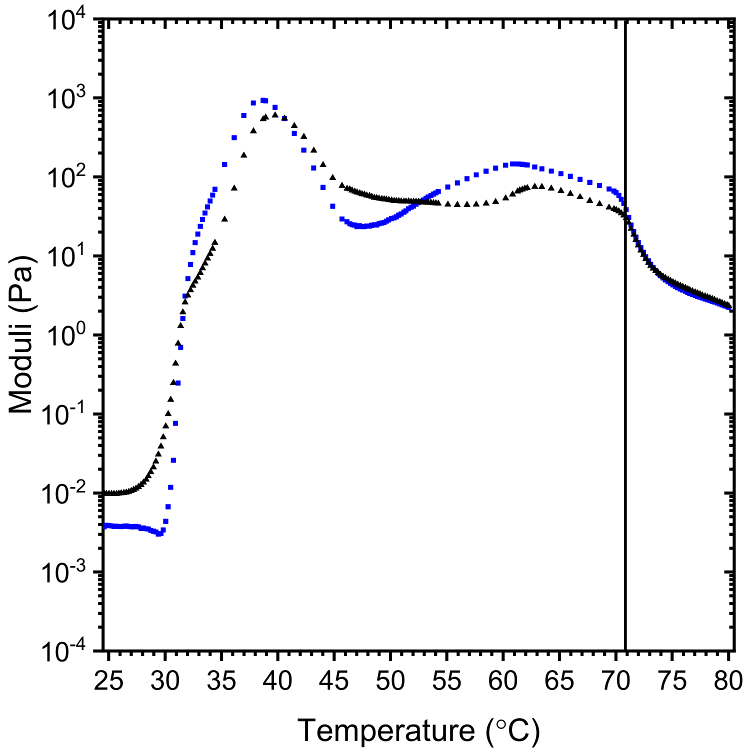

Figure S3. Temperature ramp rheological measurement on the mixture of OEGMA300$_{15}$-*b*-BuMA$_{26}$-*b*-DEGMA$_{13}$ with OEGMA300$_{10}$ at a ratio of 3:1 in phosphate buffered saline (PBS) with transitions showing up to 80 °C: storage modulus in blue squares and loss modulus in black triangles.
